# Skin Cyst: A Pathological Dead-End With a New Twist of Morphogenetic Potentials in Organoid Cultures

**DOI:** 10.3389/fcell.2020.628114

**Published:** 2021-01-12

**Authors:** Weiming Qiu, Pei-Rong Gu, Cheng-Ming Chuong, Mingxing Lei

**Affiliations:** ^1^Department of Dermatology, General Hospital of Central Theater Command of Chinese People’s Liberation Army, Wuhan, China; ^2^Integrative Stem Cell Center, China Medical University Hospital, China Medical University, Taichung, Taiwan; ^3^Department of Pathology, Keck School of Medicine, University of Southern California, Los Angeles, CA, United States; ^4^“111” Project Laboratory of Biomechanics and Tissue Repair, Key Laboratory of Biorheological Science and Technology of the Ministry of Education, College of Bioengineering, Chongqing University, Chongqing, China

**Keywords:** skin homeostasis, stem cell, multipotency, regeneration, hair follicle, tissue engineering

## Abstract

A cyst is a closed sac-like structure in which cyst walls wrap certain contents typically including air, fluid, lipid, mucous, or keratin. Cyst cells can retain multipotency to regenerate complex tissue architectures, or to differentiate. Cysts can form in and outside the skin due to genetic problems, errors in embryonic development, cellular defects, chronic inflammation, infections, blockages of ducts, parasites, and injuries. Multiple types of skin cysts have been identified with different cellular origins, with a common structure including the outside cyst wall engulfs differentiated suprabasal layers and keratins. The skin cyst is usually used as a sign in pathological diagnosis. Large or surfaced skin cysts affect patients’ appearance and may cause the dysfunction or accompanying diseases of adjacent tissues. Skin cysts form as a result of the degradation of skin epithelium and appendages, retaining certain characteristics of multipotency. Surprisingly, recent organoid cultures show the formation of cyst configuration as a transient state toward more morphogenetic possibility. These results suggest, if we can learn more about the molecular circuits controlling upstream and downstream cellular events in cyst formation, we may be able to engineer stem cell cultures toward the phenotypes we wish to achieve. For pathological conditions in patients, we speculate it may also be possible to guide the cyst to differentiate or de-differentiate to generate structures more akin to normal architecture and compatible with skin homeostasis.

## Introduction

Skin is the largest organ of the body in mammal. Along with the appendages, skin functions on regulating body temperature, defending against micro-organisms infection, and protecting the body from external harmful matters such as toxic substances and UV irradiation (Chuong et al., 2002). Skin is mainly composed of the epidermis and dermis. Epithelial-mesenchymal interactions allow development of several dynamic skin appendages such as hair follicle, sebaceous gland, sweat gland, etc., which play diverse roles in skin function (Lei et al., 2017a). Epithelial cells have apical-basal polarity and line up in a sheet. Cyst can form when epithelial cells fail to continue to the next stages of morphogenesis. Progress has been made in the investigation of how skin cyst forms and why this structure can be developed. Interestingly, in skin organoid culture, we have observed cysts form transiently, but then they undergo further morphogenesis (Tang et al., 2002; Lei et al., 2017b). This makes us contemplate whether the cysts in the skin we have encountered are not bona fide dead-ends, but are structures stuck in a morphogenetic process that misses certain morphogenetic cues. Therefore, skin cysts may be rescuable with new twists as seen in the restoration of skin cells from adult mice that lose the regenerative ability to form hairs. Here we first review the more traditional knowledge of skin cyst pathology. Then we summarize recent progress on the formation of cyst configuration during tissue engineering of skin organoid formation. We present a new perspective that the skin cyst may be turned from a morphogenetic dead-end into a morphogenetic hub if we can explore its upstream and downstream events.

## The Formation of Cyst Configurations Under Physiological and Pathological Conditions

### Classification of Skin Cyst

**FIGURE 1:**
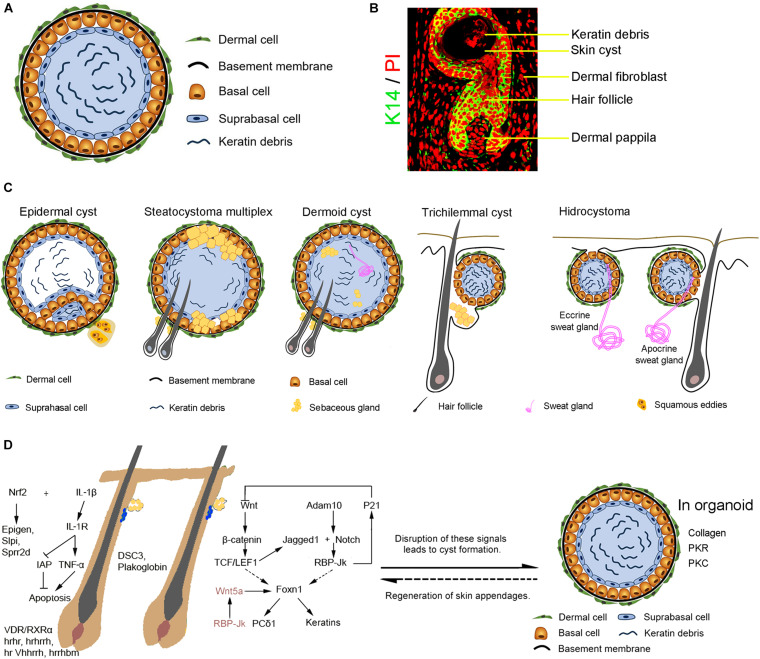
The formation of the skin cyst as a sign in pathology. **(A)** The general characteristics of skin cysts. Skin cyst consists of a cyst wall of basal cells, suprabasal cells, and cyst contents (e.g., keratin debris, sebaceous gland, sweat gland, or hair). Some skin cysts are surrounded by layers of dermal cells and a basement membrane between dermal cells and basal epidermal cells. **(B)** Relationship with hair follicles. Hair follicle induction on the cyst wall upon epithelial-mesenchymal interaction mechanism. **(C)** Different types of skin cysts. Epidermal cyst is the most common cutaneous cyst which includes the general skin cyst components. Some epidermal cyst forms squamous eddies around the cyst. Steatocystoma multiplex originated from the outer root sheath of hair follicles contains sebaceous glands and can generate hair follicles. A dermoid cyst contains can form a sebaceous gland, hair follicle, or sweat gland when interacting with dermal cells surrounding the cyst. A trichilemmal cyst originates from the outer root sheath of hair follicles and produces abundant keratin material inside the cyst. The basal cells coalesce with the suprabasal layer without a clear boundary line. Hydrocystoma forms due to disorders of the sweat gland, including the eccrine sweat gland and the apocrine sweat gland. Eccrine hidrocystoma is filled with the residue of sweat, an inner columnar cell layer, an outer myoepithelial cells layer, a basement membrane, and dermal cells. Apocrine hidrocystoma consists of lipofuscin granules contents, an inner secretory columnar layer, an outer myoepithelial layer, basement membrane, and dermal cells. **(D)** Complex signalings balance skin and appendages homeostasis and cyst formation. Knockout of *IL-1*/*IL-1R* or activation of Nrf2 disrupts skin and hair follicle homeostasis, leading to skin cysts formation. Ablation of *hrhr, hrhr^*rh*^, hr Vhhr^*rh*^*,*hr^*rhbm*^*, and *VDR/RXR*α results in hair loss and cyst formation. Disrupted expression of cell-cell adhesion molecules DSC3 or Plakoglobin results in skin cyst formation. Disruption of Wnt and Notch pathways which regulate hair growth causes the formation of skin cysts from hair follicles. Abnormality of RBP-Jk/Wnt5a signals in dermal papilla cells induces skin cyst formation. In addition, interactions between the basal epidermal cells of the skin cyst and surrounding dermal cells may result in skin appendage regeneration.

A skin cyst includes a basal epidermal layer surrounded by layers of dermal cells. The basal layer differentiates toward the inside of the cyst into the suprabasal layers which undergo terminal differentiation to produce keratin debris (Figures 1A,B). Skin cysts can be classified into five major types per cellular origins (Kaya and Saurat, 2018; Figure 1C). These include cysts with epithelial cells from infundibulum or interfollicular epidermis (epidermal cysts, comedo, milia, and eruptive vellus cysts) (Swygert et al., 2007), cysts with epithelial cells from outer root sheath (ORS) of the hair follicle (trichilemmal or pilar cysts), cysts with epithelial cells from the sebaceous duct (steatocystoma and cutaneous keratocyst), cysts with epithelial cells from sweat gland (hidrocystoma), cysts with epithelial cells from hair matrix (hair matrix cyst), and hamartomatous cysts (dermoid cyst, folliculosebaceous hamartoma and metabolizing acquired dioxin-induced skin hamartoma). Different cellular origins contribute to diverse skin cysts formation. Epidermal cyst is the most common cutaneous cysts present as nodules in the skin. The disruption of the hair follicle, implantation of the epithelium due to traumatic and penetrating injury, excessive ultraviolet (UV) radiation, infection with the human papillomavirus (HPV), medicine (e.g., imiquimod, cyclosporine, vemurafenib, etc.) (Ramagosa et al., 2008; Boussemart et al., 2013) and inherited diseases (e.g., Gardner’s syndrome) all lead to the formation of epidermal cysts. These cystic lesions are usually asymptomatic. It consists of multilayer squamous epithelia and is filled with keratinized substances differentiated by the suprabasal epithelial cells. Some squamous epithelial cells form squamous eddies around the cysts.

### Cysts Configurations Are Observed in Developmental and Physiological Conditions

Cyst formation is required in the early stage of embryogenesis and functional maintenances of organs. A blastocyst is a physical structure developed in the early development of mammals. A blastocyst contains the inner cell mass which subsequently develops the embryo and the outer layer which forms blastocele. An ovarian follicle, a spheroid cellular aggregation set, can influence stages of the menstrual cycle by secreting hormones. In every menstrual cycle, each ovarian follicle has the potential to release an egg cell for fertilization. Additionally, in adult tissues such as the thyroid gland, thyroid follicle consists of cuboidal epithelium and contents which contain colloid, thyroglobulin, and iodine. Thyroid follicles secret thyroid hormones to regulate body metabolism and growth.

### Cyst Formation Under Pathological Conditions

Cysts can be observed in almost every organ such as skin, kidney, liver, ovary, lung, joint and mammary gland, ranging from microscopic scale to several centimeters in diameter. For example, renal cysts with fluid-filled contents can be observed in a single cyst and several polycystic kidney diseases (Ferro et al., 2019). Progressive dilatation of biliary micro-hamartomas promotes the development of simple hepatic cysts in the liver (Kimura et al., 1978; Jung et al., 2012). Ovarian cysts including functional cysts and non-functional cysts are usually filled by fluid or semiliquid material (Bottomley and Bourne, 2009). Ganglion cyst is often formed next to a joint and usually filled with excess joint fluid (Hsu et al., 2007). Breast cyst is a fluid-filled sac, and it is necessary to ensure whether breast cyst is benign or malignant. Cyst is also present in fibrosis which is an inherited disease. Cystic fibrosis can cause progressive damage to the respiratory system and chronic digestive system (Castellani and Assael, 2017).

## Pathogenesis of the Skin Cyst

### Imbalance of Skin Homeostasis Leads to Cyst Formation

Skin cysts can form due to mistakes during skin development. For example, variants in PLCD1 are detected in families with multiple trichilemmal cysts (Shimomura et al., 2019). Dermoid cysts form due to the sequestration of ectodermal tissues during embryonic closure. These cysts originated from ectoderm locate in the subcutaneous tissue, but still maintain the multipotency to develop fully differentiated ectopic structures, such as nails and dental, cartilage-like, and bone-like structures. This suggests that the formation of cysts might be a self-protection response to mistakes during skin development.

Skin is constantly subjected to insults such as UV radiation, toxin, invasive pathogens, etc (Lei and Chuong, 2018). In response to these insults, skin epidermis initiates a rapid innate immune response by producing chemokine, antimicrobial peptides, and Toll-like receptors (Selleri et al., 2007; Reithmayer et al., 2009; Nagao et al., 2012). Skin keratinocytes produce IL-1β in response to UV radiation and infection (Dai et al., 2017). IL-1 receptor (IL-1R) or IL-1β knockout mice develop multiple epidermal cysts when receiving chronic UVB exposure (Kulkarni et al., 2017). IL-1β signaling mediates UV-induced proinflammatory responses (Maelfait et al., 2008) and promotes UVB-induced apoptosis through driving TNF-α release and down-regulating inhibitor of apoptosis proteins (IAP) (Kothny-Wilkes et al., 1999). Knockout of IL-1R blocks the expression of TNF-α and promotes keratinocytes to be resistant to UVB-induced apoptosis, leading to the disruption of skin homeostasis and skin cysts formation. Transcription factor Nrf2 plays an important role in protecting skin from reactive oxygen species which are induced by the harmful insults from microorganisms, UV light, and toxic chemicals (Schafer et al., 2010; Tan and Wahli, 2014). Nrf2 activation in keratin 5 (K5)-positive skin epithelia leads to hair loss, infundibula dilatation, sebaceous gland enlargement, and cyst formation, with upregulation of Epigen, Slpi, and Sprr2d in the cyst (Tan and Wahli, 2014).

The inflammatory response to the grafted epidermal tissues during implantation can be important in the formation of skin cysts. To protect tissues from stimuli such as pathogens infection or damage in cells, inflammatory responses involving immune cells, blood vessels, and molecular mediators are mobilized to eliminate the necrotic cells and tissues to facilitate tissue repair (Ferrero-Miliani et al., 2007). Thus, after the implantation of ectopic epidermal tissues, inflammatory response can be activated to wrap these ectopic epidermal tissues, leading to cyst formation.

Cell-cell adhesion is the basis for forming tissue integrity. Conditional knockout of Desmocollin3 (Dsc3) in the skin epithelium destroys the cell-cell adhesion, causing intra-epidermal blistering, and cyst formation in the hair follicle (Chen et al., 2008). Another cell adhesion molecule Plakoglobin as a cytoplasmic constituent of the desmosome is involved in the intracellular signaling events essential for epidermal differentiation. Specific expression of N-terminally truncated plakoglobin in epidermis results in the formation of additional hair germs, hyperplastic hair follicles, dermal cysts, and even non-invasive hair follicle tumors (Teuliere et al., 2004). The epithelial cells of the cysts are derived from precortex and hair matrix cells and show hair follicle or epidermal characteristics by molecular characterization.

### Different Types of Hair Follicle-Derived Skin Cysts Help Reveal the Molecular Control of Cyst Formation

A hair follicle is a complex micro-organ with multiple cellular components including the bulge, ORS, inner root sheath (IRS), hair bulb, and dermal papilla. Homeostasis of the hair follicle is maintained by the coordination of extracellular matrix signaling, autocrine signaling, paracrine signaling, systematic signaling, etc (Chueh et al., 2013). Abnormality in hair follicle development and regeneration often leads to cyst formation (Vidal et al., 2005).

Sonic Hedgehog (Shh), Transforming Growth Factors β (TGFβ), and Bone Morphogenetic Protein (BMP) signaling pathways are required in hair follicle morphogenesis and hair follicular growth (Adam et al., 2018; Lim et al., 2018; Rahmani et al., 2018). Specific deletion of Shh receptor Smoothened in skin epithelium causes a transformation of ORS to epidermis-like structure, hair loss, and cyst formation (Gritli-Linde et al., 2007). Specific deletion of TGFβ mediator Smad4 in basal skin epithelial cells results in epidermal and follicular hyperplasia, progressive hair loss, and cyst formation (Yang et al., 2005). Cyst formation is also observed in skin epithelia-*Bmpr1a* knockout and activation mice (Kobielak et al., 2003, 2007; Yuhki et al., 2004).

Wnt/β-catenin signaling largely influences hair follicle stem cells (HFSCs) behaviors including activating HFSCs during regeneration and promoting their differentiation during hair growth (Rabbani et al., 2011; Lei et al., 2013, 2014). Deletion of β-catenin in basal epidermal and hair follicular keratinocytes causes epidermal hyperplasia and cyst formation (Huelsken et al., 2001). K15 and integrin β1, markers of HFSCs, are expressed in the cyst wall, suggesting that the multipotency of epidermal stem cells may be partially maintained through the formation of cyst after epidermal deletion of β-catenin. Enhanced Wnt signaling also causes not only tumorigenesis but also skin cyst formation (Gat et al., 1998; Hassanein et al., 2003). Overexpression of truncated LEF1 without the β-catenin interacting domain in keratinocytes suppresses hair differentiation but induces sebaceous gland differentiation and causes cyst formation beneath the bulge region. These cysts exhibit a multipotency of epidermal stem cells or sebaceous gland stem cells (Merrill et al., 2001), suggesting that LEF1 may interact with transcriptional inhibitors and promote the progeny of HFSCs to transform into a population of cells with the multipotency of epidermal stem cells or sebaceous gland stem cells.

Notch signaling pathway is important in maintaining the homeostasis of hair follicles and epidermis. Specific deletion of Notch pathway genes such as *Jagged 1, Notch1, Notch2*, or *RBP-J*κ in skin epithelia blocks the differentiation of IRS, induces the differentiation of ORS cells besides HFSCs, and causes the skin cyst formation (Demehri and Kopan, 2009). Moreover, conditional knockout of Adam10 in skin epithelia results in impaired expression of Notch pathway target genes *Hes* and *Hey* and causes hair loss, epidermal hyperproliferation, and epidermal cyst formation (Weber et al., 2011). Disruption of mesenchyme-derived signals also causes skin cyst formation. Deletion of CSL/RBP-Jκ, the effector of the canonical Notch pathway in dermal papilla cells and dermal fibroblasts causes the degeneration of hair follicles into epidermal cysts, with high levels of interfollicular marker expression (Hu et al., 2010). Mesenchymal deletion of RBP-Jκ results in a decreased expression of dermal papilla specific signatures, such as Wnt5a, Fgf7, Fgf10, and Noggin (Rendl et al., 2005). Hair follicle reconstruction assay by injecting *Wnt5a*-deficient dermal papilla cells and normal keratinocytes into the nude mice skin shows the development of skin cysts rather than hair follicles (Rendl et al., 2005). Besides, Notch1 can activate the *Wnt5a* promoter by RBP-Jκ (Rendl et al., 2005), suggesting that Wnt5a is a primary target gene of the activated RBP-Jk to control normal hair follicle development or cyst formation. Functioning downstream of Wnt5a, Foxn1 directly activates the Notch1 promoter by binding Notch1 promoter in mice (Cai et al., 2009), to regulate hair differentiation. This suggests that the Notch-Wnt5a-Foxn1 mesenchymal-epithelial signaling module functions as a positive feedback to regulate hair differentiation. Besides, deletion of Phospholipase C(δ1 (PCδ1), one of the target genes of Foxn1, also causes cyst formation (Nakamura et al., 2003).

Suppresion of histone modification-related enzymes can lead to the failure of hair follicles to regenerate properly, and therefore turn the hair follicles into cysts. For instance, *Hdac1* maintains the homeostasis of epidermis and hair follicles. Epithelium-specific knockout of *Hdac1* in mice causes hyperkeratosis, hair follicle dystrophy, extensive alopecia, and epithelial cyst-like structures (Hughes et al., 2014). However, Krt14 and Trp63 which mark the epidermal stem cells are expressed in the whole cyst wall. This suggests that hair follicle progenitor-derived cells retain certain multipotency properties of epidermal stem cells. The degeneration of hair follicles into epidermal stem cells is also present in other epidermal cysts (Demehri and Kopan, 2009; Kulkarni et al., 2017; Yang et al., 2020).

Perturbation of several genes or signaling pathways that are necessary for the skin homeostasis and hair follicle formation results in epidermal cysts formation. These genes include hairless (hrhr), hairless-rhino (hrhrrh), rhino (hr Vhhrrh), bald Mill Hill (hrrhbm) (Mann, 1971; Brancaz et al., 2004), Vitamin D receptor (VDR) (Keisala et al., 2009) and RXRα (Li et al., 2001), etc.

Mapping the molecular mechanisms by which these signaling pathways interact with each other to control the hair follicle homeostasis or skin cyst formation requires further bioinformatics approaches and functional tests. At least known studies have revealed that Wnt and Notch pathways are largely involved in this molecular regulatory network (Figure 1D).

## The Formation of Cyst Configuration in Organoid Cultures

Recent studies on organoid formation demonstrate that the cyst formation is a common and essential process to generate the mini-organs (Bredenoord et al., 2017; Dutta et al., 2017; Smith and Tabar, 2019; Lee et al., 2020). Most organoids are derived from embryonic stem cells, induced pluripotent stem cells (iPSCs), or primary cultured cells. The cells are guided or self-organize to form a cyst-like structure in most types of organoid culture before induction of the desired tissues/organs (Rossi et al., 2018). Some cells (like dissociated endothelial) form tubes when they are cultured. Different from formation of a tube which is a long hollow cylinder structure, involving tissue mechanics of ECM and cytoskeleton. It would be interesting to identify the driving forces of each arrow in the morphospace in Figure 1B, whether it is biochemical or biophysical based. We think this would be the challenge of next step in organoid study, to be able to guide the formation of desired tissues for application. Progress has been made in identifying the novel characteristics of the cyst formed *in vivo* and *in vitro*. Growing evidence shows that the cyst structure retains certain multipotency to self-renew and differentiate, and *de novo* regenerate mini-organs resembling those formed during the developmental processes. Cyst forms budded structure during intestinal organoid development, with involvement of fate-specific changes in osmatic and actomyosin forces, as well as Yap signaling pathway activation (Serra et al., 2019; Yang et al., 2020). This endows the cysts not only as a pathological structure but also with the potential to study how cells retain multipotency to generate new organs *in vivo* and *in vitro*.

Tissue-engineered skin represents a useful strategy for the treatment of skin injuries. Primary cultured skin cells, iPS-differentiated skin cells, embryonic stem cells-differentiated skin cells, etc., are used to generate fully functional skin with appendages formation. Accompanying with fully functional skin formation during the explant transplantation into the nude mice is the formation of skin cysts, which express the epidermal stem cell markers. Appendages such as hair follicles not only form from the regenerated skin but also the skin cysts (Zheng et al., 2005; Balana et al., 2015; Lei et al., 2017b; Lee et al., 2020).

**FIGURE 2:**
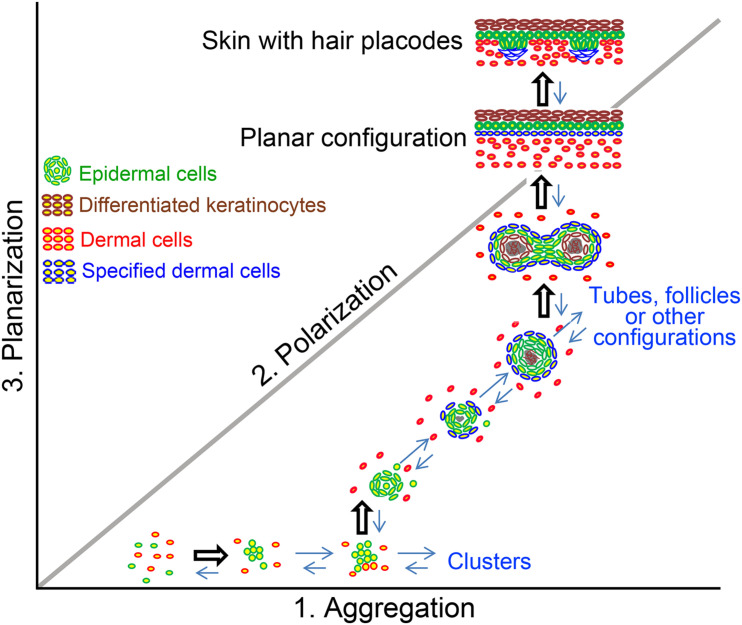
The formation of cyst configuration in skin organoid culture. A morphospace of potential epidermal and dermal cell configuration is shown. Pattern diagram showing possible multicellular configurations during newborn skin cells organoid culture. Dissociated cells undergo a series of morphological transitions including aggregation, polarization, coalescence, and planarization, to form hair primordia [The figure is from Lei et al. (2017b)].

Skin cyst formation also occurs when pluripotent stem cells are cultured *in vitro*, which is termed skin organoid culture (Lei et al., 2017b; Lee et al., 2018, 2020). The *in vitro* cultured organoids can form the fully functional skin with epidermis and dermis structures, as well as with regeneration of skin appendages such as hair follicle (Figure 2; Lei et al., 2017b; Lee et al., 2018), sebaceous gland (Feldman et al., 2019) and sweat gland (Diao et al., 2019). This indicates that though skin cyst is an abnormal structure to the organism, the formation of the skin cyst can be the other way for the tissue to retain sufficient multipotency. And importantly, the process of the cyst formation during the cell self-organization *in vitro* is required for further tissue regeneration. The reason why cells prefer to choose this more complex way to form fully functional skin requires further investigation. However, one possibility is that the easiest way for one to reach the destination from the beginning may not be through a straight line but by consuming their lowest energy. Thus, the skin cells may use the same strategy to form fully functional skin by forming the skin cyst to maintain their multipotency. Indeed, adult cells with declined regenerative ability can be reactivated to form skin organoid through cyst formation by applying certain factors during culture and regenerate hair follicles upon regeneration (Lei et al., 2017b).

## Conclusion and New Possibilities

Here we show cyst form from the progenitor/stem cells who find their route to maintain multipotency and can regenerate the original tissues under pathological and skin organoid culture conditions. Cysts in pathological conditions are formed from complex tissue architectures by making closed sac-like tissue structures that contain simple or complex tissues depending on their origin and mutation type, with complex molecular regulatory networks largely involving Wnt and Notch pathways. They also tend to form during renewal conditions such as hair cycling. They failed to form complex structures and get “stuck” in the cyst stage. Organoids first form cysts with relatively homogeneous progenitors and tissue structures, and then start developing more complex tissue architectures with cell differentiation and tissue patterning, with the involvement of extracellular matrix and protein kinases C and R (Figure 1D). These lead to a new look of the skin cysts as potential new therapeutic possibilities.

However, several outstanding questions regarding skin cyst formation remain. For example, how to guide cyst in organoid cultures to continue morphogenesis? This can be achieved by modifying the molecular profiles of the cyst cells. The configuration may also be changed by 2-dimensional or 3-dimensional culture conditions. Activation or de-stabilization of the cyst can also be achieved by altering the physical properties of the surrounding environment, by affecting the affinity of ECM and cyst epithelial cells. What happens when the cyst configuration de-stabilization? Is it the loss of apical-basal cell polarity? Realignment of cell adhesion and neighboring cellular partners? Does the rigid structure enter a more fluid state similar to those observed in epithelial-mesenchymal transition?

With the organoid cultures as a platform to investigate the mechanism by which skin organoids undergo a morpho-space development, from aggregate to skin cyst, then to a planar skin with hair regeneration will help answer these questions. As researches on the topic of skin biology continue, understanding the control of cyst configuration, we hope to provide a theoretical basis for understanding the pathogenesis of skin cysts, improving the therapeutic methods of skin cysts, and producing more functional skin organoids for tissue regeneration.

## Data Availability Statement

The original contributions presented in the study are included in the article/Supplementary Material, further inquiries can be directed to the corresponding author/s.

## Author Contributions

C-MC and ML contributed to the conception and design of the manuscript. WQ, P-RG, C-MC, and ML wrote the article and approved the final version. All authors read and approved the final manuscript.

## Conflict of Interest

The authors declare that the research was conducted in the absence of any commercial or financial relationships that could be construed as a potential conflict of interest.

## Abbreviations

iPSCs: induced pluripotent stem cells
ORS: outer root sheath
UV: ultraviolet
HPV: human papillomavirus
IL-1R: IL-1 receptor
IAP: inhibitor of apoptosis proteins
MADISH: metabolizing acquired dioxin-induced skin hamartomas
VDR: Vitamin D receptor
Dsc3: Desmocollin3
HFSCs: hair follicle stem cells
IRS: inner root sheath
APC: adenomatosis polyposis coli
GSK3: glycogen synthase kinase 3
CK1 α: casein kinase 1 α
Shh: Sonic Hedgehog
IC: intercellular domain
Adams: A disintegrin and metalloproteases
PC δ 1: Phospholipase C δ 1.
